# Deep mapping gentrification in a large Canadian city using deep learning and Google Street View

**DOI:** 10.1371/journal.pone.0212814

**Published:** 2019-03-13

**Authors:** Lazar Ilic, M. Sawada, Amaury Zarzelli

**Affiliations:** 1 Laboratory for Applied Geomatics and GIS Science (LAGGISS), Department of Geography, Environment and Geomatics, University of Ottawa, Ottawa, Canada; 2 l’École nationale des sciences géographiques (ENSG-Géomatique), Paris, Champs-sur-Marne, France; Universidade Estadual de Maringa, BRAZIL

## Abstract

Gentrification is multidimensional and complex, but there is general agreement that visible changes to neighbourhoods are a clear manifestation of the process. Recent advances in computer vision and deep learning provide a unique opportunity to support automated mapping or ‘deep mapping’ of perceptual environmental attributes. We present a Siamese convolutional neural network (SCNN) that automatically detects gentrification-like visual changes in temporal sequences of Google Street View (GSV) images. Our SCNN achieves 95.6% test accuracy and is subsequently applied to GSV sequences at 86110 individual properties over a 9-year period in Ottawa, Canada. We use Kernel Density Estimation (KDE) to produce maps that illustrate where the spatial concentration of visual property improvements was highest within the study area at different times from 2007–2016. We find strong concordance between the mapped SCNN results and the spatial distribution of building permits in the City of Ottawa from 2011 to 2016. Our mapped results confirm those urban areas that are known to be undergoing gentrification as well as revealing areas undergoing gentrification that were previously unknown. Our approach differs from previous works because we examine the atomic unit of gentrification, namely, the individual property, for visual property improvements over time and we rely on KDE to describe regions of high spatial intensity that are indicative of gentrification processes.

## Introduction

Working in London, UK, Glass (1964), coined the term ‘gentrification’ to describe the process whereby the working class is displaced by upper classes (the gentry) in urban space [1]. Class displacement resulting in gentrification has been observed in many western cities [2–4]. However, the conceptualization of gentrification has expanded over time to focus on the driving factors of the process such as culture and consumption [5–7], political economy and production [8–11] and, indeed, the definition has extended to include different groups of gentrifiers ranging from marginal [12] and middle class [7] to the working class [13]. Current thought on gentrification is intersectional, examining how various discourses are implicated, such as bodies [14] or industrial spaces [15] and, as such, gentrification has been identified as taking place where it was once not seen as possible [16]. Recent years have witnessed an expansion of the topic into tourism [17], planning and policy impacts [18], environment [19,20], and many other specialized topics [21–26]. The Economist tweeted a quote by Dyckhoff claiming that gentrification is “the most significant force in Western cities in the second half of the 20^th^ century.” [27,28]

When a higher socioeconomic class subjugates an existing urban locale, there are visible changes to the building stock. Visually/aesthetically, housing stock re-investment is the most acute indicator of gentrification [29]. As indicators of gentrification, visible changes to building stock can manifest in several ways. Given that most studies on gentrification are concerned with the discourse, politics and socio-cultural aspects that shape the gentrification process, the characteristic aesthetic changes taking place are often ignored even though they are the primary external indicator of the process. As a result, there is a literature gap in measuring and quantifying the visual expression of the gentrification processes, probably because as Hammel and Wyly [29] rightly note, the visual expressions of gentrification processes are difficult to observe and measure.

In short, property improvement over time is an important sign of gentrification and is perhaps one of the most important yet overlooked indicators of the process. Jager [30] has demonstrated that various types of renovation and restoration initiatives serve to assert social rank, because housing confers the attributes of social status and prestige. Redfern [31] notes that gentrification cannot emerge if property improvement is not present. As Gentrification is “the production of urban space for progressively more affluent users” [32], it is fair to assume that a number of visual indicators reveal this process in a given location.

Some scholars have analyzed gentrification based on the direct auditing of the built environment. Direct auditing methods are those that require people to manually examine the GSV images (or the real world) for perceptual attributes [33,34]. Using direct auditing methods, Hammel and Wyly ([29] Table 1) sought visible signs of reinvestment in both single family homes and multi-unit buildings. Features observed in single unit homes included property repainting, changes in the degree of ornamentation, reconstruction of porches, steps, window replacement, fences, and so forth. Meanwhile, multi-unit housing would have some other visible features such as entryway and signage changes, porch furniture, new brickwork/siding, and the like (Hammel and Wyly 1996, 250–251).

**Table 1:** Results of model after fine-tuning.

	# of Training batches	Number of epochs per batch	Iterations with batch size of 24	Validation accuracy	Test accuracy
Fully connected layers (SCNN-FC)	8	50	70,000	92.2	-
Fully connected layers and top 4 convolutional (SCNN-FC-4)	8	50	70,000	94.6	-
FC + top 8 convolutional layers (SCNN-FC-8)	4	50	35,000	95.2	95.6%

Heidkamp and Lucas [35] examined additional visual changes in gentrification processes, including non-residential upgrades, the renovation of commercial space, improvements to the street infrastructure, and even the noticeable presence of vehicles that represent upgraded transportation. Streets were sometimes redesigned to imitate cobblestones of the past. The transformation of commercial space is noteworthy because it too gentrifies and caters to consumer preferences of the higher class [36].

The aforementioned studies identify numerous visual impacts that indicate gentrification to properties. However, technological availability limited those studies to small spatial domains (a few city blocks) because of the onerous time demands that primary observational work requires [35]. It is useful to note that Google Street View (GSV), by itself, reduces the time required for direct auditing research by eliminating field logistics in the real-world and this leads to as much as 50% time savings when collecting data [33,37].

Google Street View (GSV) data provides opportunities to analyze urban space for visual gentrification-like changes at the atomic level of the gentrification process: the property level—the smallest spatial unit upon which a gentrification process can select/act upon—and at frequencies higher than the bi-decadal or decadal census. Moreover, with GSV, potentially every urban structure can be examined for visual changes through time (back to 2007 in most cases in the United States and Canada). While GSV yields large quantities of data, manual examination of each individual structure’s GSV images over time for the hallmark visual indicators of gentrification is untenable in a large urban center. However, recent advances in machine learning and computer vision can be leveraged to examine each structure over time in a large urban center to produce detailed maps of visual changes. Machine learning applied to GSV imagery has the potential to revolutionize the study of the spatial expression of the gentrification process.

The intention of this paper is not to debate the potential benefits or drawbacks of gentrification but rather to assess the potential of machine learning to detect the visual process of gentrification. Herein we propose a machine learning approach using deep convolutional neural networks to examine hundreds of thousands of GSV images to detect the associated visual impacts that are characteristic of urban renewal and change.

## Deep mapping and the built environment

### Google Street View (GSV) in auditing the urban environment

GSV has been used to uncover intricate details in neighborhoods using direct auditing methods. For example, research in the UK suggests that GSV is an accurate tool for ascertaining if a neighborhood is struggling [38]. GSV has also been used to virtually evaluate physical environmental characteristics along cycling routes [39]. In general, the use of GSV has a longer history within the development of systematic social observation instruments (SSOIs) applied to the urban environments [40–45].

Few gentrification studies have been conducted using Google Street View (GSV) as a data source. A study by Hwang and Sampson [46] was one of the first to use GSV to measure gentrification in some manner. Street-level panoramic imagery offers a unique perspective because the update interval is relatively short compared to other data sources used in gentrification studies such as census data. GSVs of a property are separated by time intervals of between one to three years. Such imagery can be used to understand short-term gentrification in addition to detecting the process in the initial stages.

### Machine mapping and deep mapping

The production of spatially detailed maps illustrating gentrification processes across entire cities is beyond the ability of most research that uses GSV in direct auditing methodologies. There have been limited studies that have used machine learning techniques with GSV for the sole purpose of mapping perceptual attributes of urban spaces. We refer to the general approach of using machine learning techniques such as computer vision algorithms (GIST, Texton histograms, etc‥) applied to street-level imagery for mapping the perceptual environment as ‘machine mapping’. Within machine mapping, we propose “deep mapping” to denote the use of deep learning approaches that employ convolutional neural networks (CNN) trained with street-level imagery to produce spatial data that is used for mapping or within other geocentric analyses.

#### Machine mapping

Salesses, Schechtner, & Hidalgo (2013) produced the first large-scale machine mapping study using GSV. They mapped the perception of safety in select US cities. They used crowd-sourced data from over 7000 individuals from 91 countries who responded to a website with a game that showed pairwise comparisons of GSV images from Boston, New York, Linz and Salzburg and asked the question “Which looks safer?”, the left or right image. Given that each image was compared to numerous others in the online game, the authors were able to compute a ‘Q-score’ for each image that provided a relative ranking of a each of the images along the perceptual dimension considered. The crowd-sourced dataset they produced was made available and called Place Pulse 1.0 [47].

Naik et al. [48,49] subsequently used the Place Pulse 1.0 dataset to compute a ‘Streetscore’ for 21 US cities. They used the Trueskill ranking algorithm to rank the images in the Place Pulse 1.0 dataset. Using those rankings together with corresponding state-of-the-art feature vectors (GIST, HOG2x2, SIFT etc.), they employed support vector regression to predict the perceived Streetscore for over one million GSV images. Their study produced the first high resolution street-level maps derived from computer vision and machine learning techniques.

Naik et al. [50] used the Streetscore to predict the ‘Streetchange’ of over one and a half million GSV images in several US cities. The change in Streetscore values for a given pair of images at successive time intervals at the same location was used to compute the ‘Streetchange’. They only included the years of 2007 and 2014. A positive Streetchange score indicated that the perception of visual safety of a street increased between two time periods. This study was innovative in that it applied machine learning and computer vision techniques to GSV images to map out physical improvements at fine spatial scales in order to provide a basis to test three theories of urban change. Porzi et al. [51], however, found that predicting the perceived safety level between two GSV images, the process upon which Streetchange is based, is significantly improved using CNN when compared to traditional techniques that are based on feature extraction (GIST, HOG etc‥) and ranking support vector regression algorithms.

#### Deep mapping

Within machine mapping studies, there have been several applications of deep mapping. For example, to better understand which features (e.g., canal, valley, trees) lead to scenic beauty, [52] used a CNN to extract features from images that had been rated for their scenicness through crowd-sourcing. They then used a CNN to predict the scenicness of un-rated images to produce a map of scenicness across London, UK.

In 2017, Gebru et al. [53] used a CNN and 50 million GSV images to classify and map 22 million vehicle types in the US and compare the results to census data. Liu et al [54] used a CNN and street-level images taken by Baidu (China’s equivalent to GSV) to map out the quality of buildings across Beijing, China. Similarly, a team has combined GSV images and Google Maps 3D as input to a CNN in order to map building frontage quality on a structure by structure basis across London, UK [55]. More recently, CNNs have been utilized to classify buildings into semantic categories in several cities using Street View images [56].

Dubey et al [57] developed the Place Pulse 2.0 dataset containing over one million pairwise comparisons of GSV images, from several countries, along eight perceptual dimensions. In contrast to Naik et al. (2014, 2017), Dubey et al. [57] used a deep mapping approach and trained a Siamese fully-convolutional neural network to both predict pairwise comparisons for perceptual attributes as well as a second CNN with fully connected top layers to rank images according to a given perceptual attribute. The prediction accuracy of Dubey et al. [57] was ~73%, largely because Place Pulse 2.0 data is heterogenous, derived from pairwise image comparisons among 28 different countries. People from different cultural milieus will introduce ontological and semantic inconsistencies in the dataset and this ultimately affects the generalization potential of a CNN model. However, the biases in the dataset may be relatively minor. [47]), found little bias due to age, gender or location (US vs. non-US) in the Place Pulse 1.0 dataset but that was relatively small compared to Plase Puse 2.0. To improve predictability of deep mapping models, Blečić et al. [58] collected over 17,000 GSV images in Italy and, with each being rated on a perceived ‘walkability’ scale of 1–5, they trained a CNN to rate GSV images and map their walkability scores for several Italian cities. The authors achieved accuracy of 78% and 99% (for ‘1-class-off’). The results of Blečić et al. (2018), suggest that deep mapping applications may achieve improved prediction performance when trained with regionally consistent GSV datasets.

The aforementioned studies demonstrate that machine learning techniques applied to GSV imagery provide a monumental leap in our ability to classify and map urban space for several kinds of perceptual/semantic attributes. For example, a machine should be able to learn how to compare two sequential images and identify if a gentrification-like visual change has taken place. By mapping the locations of GSV sequences that contain a visual change, gentrification-like visual changes can be mapped at highly detailed spatial scales over large spatial domains.

## Methodology

Herein, we adopt a deep mapping approach and employ a Siamese-CNN (SCNN) to detect improvements in the frontage quality (building structure plus the front of the property) of individual properties using imagery through time from GSV in Ottawa, Canada. Our approach differs from previous works because we examine the atomic unit of gentrification, namely, the individual property, for visual improvements over time that are indicative of gentrification processes. Because individual properties can undergo a visual gentrification-like change anywhere in geographic space, the SCNN becomes a detector and we rely on mapping the SCNN’s detections to identify clusters of visual changes in space. Our reasoning is as follows: If there is a spatial concentration (a high intensity) of increasingly positive changes in the physical appearance of several properties in close proximity, then this spatial concentration is indicative of a gentrification-like process. However, any given region of high spatial intensity cannot definitively confirm gentrification as the causal factor: to do so would require information on the socioeconomic and cultural changes within and around that space. Nevertheless, as discussed in the introduction, housing stock re-investment and the visual changes that ensue are an acute indicator of gentrification [29]. Therefore, while not confirmative, regions exhibiting a high intensity of visual property upgrades provide a spatial hypothesis that can be tested by examination of other factors implicated in the gentrification process.

### Study area

**Fig 1:**
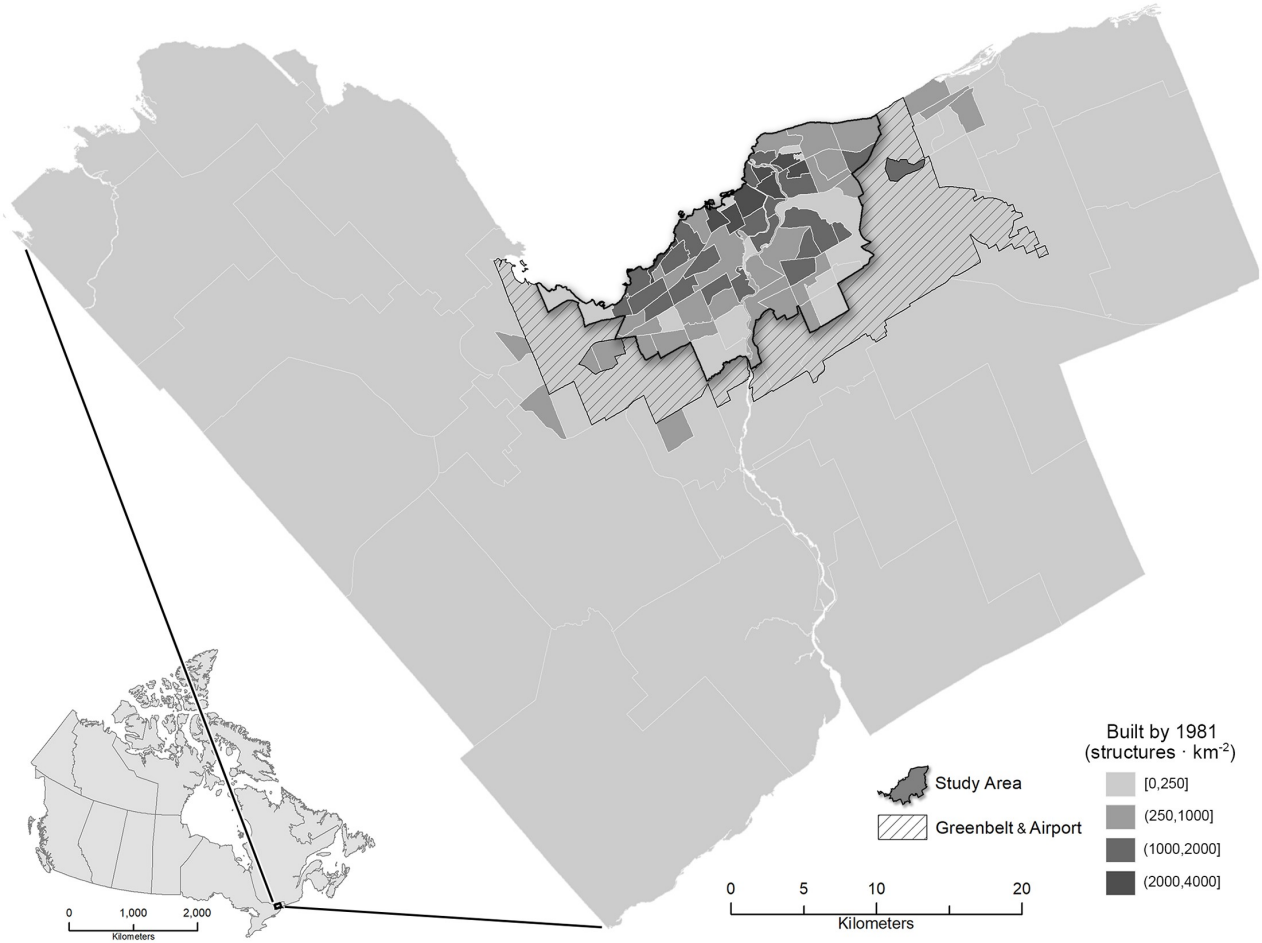
The study area contains the oldest building stock in Ottawa and is within the Greenbelt.

The City of Ottawa is Canada’s capital and has a population of 934,000 (metropolitan, 1.32 million) in 2016 [59]. Within the city boundaries, the region containing the oldest building stock (Fig 1) is most likely susceptible to gentrification pressures. The region of the city that falls within the ‘Greenbelt’—an agricultural land use zone intended to stop urban sprawl—contains the highest densities of pre-1981 structures and thus defines our study area. For every property within the study area, the SCNN will search for gentrification-like visual changes in the GSV time sequence of images. Moreover, the study area is the only contiguous region that contains GSV images beginning in 2007 (Section C in S1 Appendix).

### Training data

Google Maps Application Programming Interface (API) was used to access panoramas (full 360° hemispherical views) of every property within the study area as well as within suburban regions that were adjacent to the greenbelt. When this work was completed, prior to July 16th, 2018, our use of GSV complied with the Google Maps APIs Terms of Service. The City of Ottawa building footprints and street network [60] were used to calculate a vector perpendicular to the street and looking at each building structure. The origin (longitude / latitude), look direction and field of view were fed to the Street View API to clip a GSV image from all the time-stamped panoramas at each location. There were 157,303 properties at which panoramas were available, leading to a final count of 593,723 clipped GSV images (Figure A(A) in S1 Appendix). We then selected a subset of 9462 properties, containing 16,224 individual images, from several neighbourhoods for training the SCNN (Figure A(B) in S1 Appendix).

**Fig 2:**
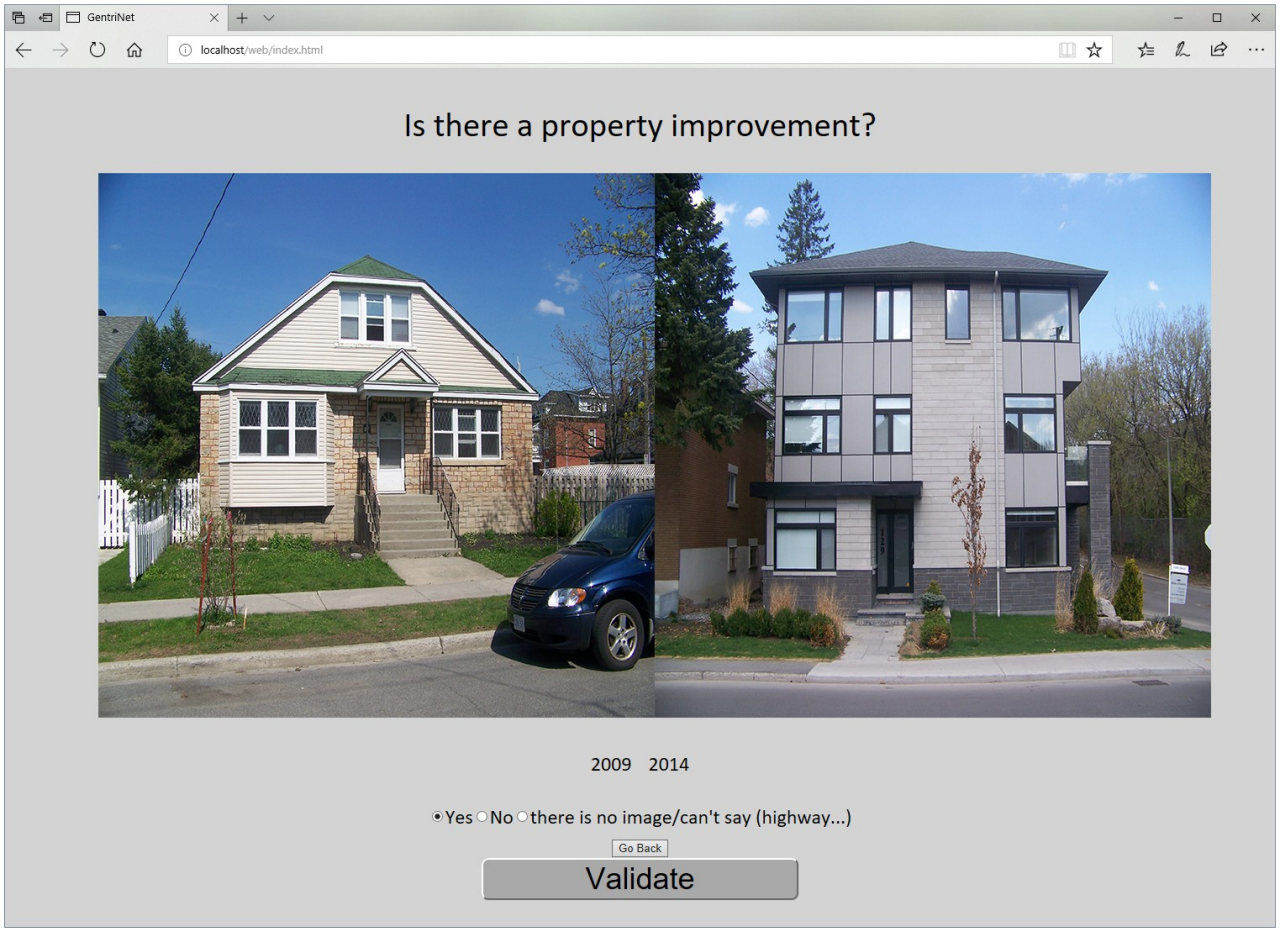
Training data collection web interface. To comply with CC-BY copyright the photos are the author’s and presented for illustrative purposes. The photos are similar to those from GSV.

The GSV dataset used to train the model was comprised of 16,224 paired image comparisons, of which, 1307 (at 1275 unique locations) were positive and 14,917 pairs (at 8594 unique locations) were negative for a gentrification-like visual change. The sum of unique locations for positive and negative physical changes (*n* = 9869) is greater than the sum of unique locations for the entire training set (*n* = 9462) because, at a given location, there can be multiple pairs that have a negative for change or positive for change. Following the data collection protocol of Place Pulse 1.0 [47] and Place Pulse 2.0 [49,50], we created the training dataset using a webpage in which a property (long,lat) was selected and the user was shown two sequential GSV images within the sequence of time-stamped images at that location and, for each pair, asked to respond to the question: ‘Is there a property improvement?’ (Fig 2). The authors of this paper undertook the comparisons, with it being understood that we were concerned with property improvements that are identifiable as gentrification-like visual changes. A gentrification-like visual change to a property is anything that provides evidence of a significant reinvestment in the property being viewed, including such things as the removal of an older house and construction of a new house, significant updates to the façade of an existing structure, or a clear improvement in landscaping and other relevant visual changes that are suggestive of the visual cues of gentrification including those identified by [29,35]. Unlike the Place Pulse dataset, our line of investigation was centered on the atomic unit of gentrification: the individual property, which includes the building structure and frontage together rather than a default GSV camera orientation. Thus, using the webpage (Fig 2), we were able to record when a GSV image pair exhibited a gentrification-like visual change between each successive image for the same property. The result of each comparison was then saved in a file, recording the unique GSV assigned image identifier (panoid) for the left image, right image and yes or no for property improvement (Section B in S1 Appendix).

The training dataset was subsequently randomly shuffled and 40% was held back for model validation (n = 3245 pairs) and testing (n = 3245 pairs). The remaining 9734 pairs were used for model training.

### Siamese model

**Fig 3:**
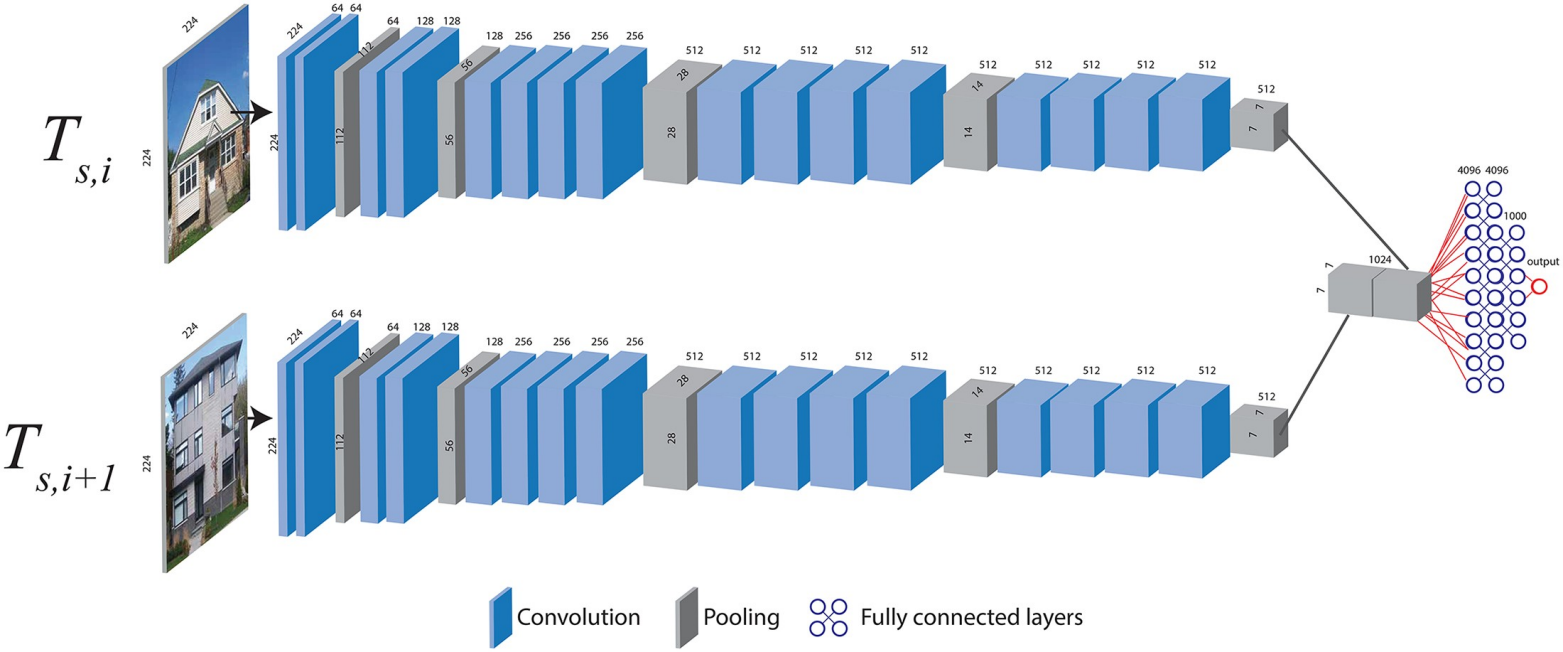
Siamese CNN architecture using two VGG19 branches. The top branch convolves the first image, *T*_*s*,*i*_, in the sequence of images at a unique geographic location *s*, (*T*_*s*_ = {*T*_*s*,*i*_: *i* ∈ *T*_*s*_}, *s* = (*x*, *y*)), and the bottom branch convolves the second image *T*_*s*,*i*+1_ in the sequence. The last pooling block in each branch are concatenated and flattened before being fed to a set of three fully connected layers to produce the output probability using a sigmoid activation. The numbers along the sides of each convolutional layer represent the tensor dimensions (columns and rows), and the numbers across the top of the layers represent the tensor filter block’s depth. Photos are the author’s and are presented for illustrative purposes.

We trained a Siamese CNN (SCNN) to detect the visual improvements to a given property between two successive GSV images (Fig 3). We programmed the SCNN in python using the Keras library [61] and a tensorflow backend (Section B in S1 Appendix). An excellent introduction to convolutional neural networks can be found in the book ‘Deep Learning With Python’ by Francois Chollet [62].

To train the Siamese model (Fig 3), a transfer learning approach was employed [63,64], whereby the two VGG19 [65] convolutional branches (Fig 3) were initialized using weights trained on the ImageNet [66] challenge database [67]. These VGG19 model weights are the result of training on 1.3 million images with 1000 categories [65]. Because the ImageNet weights were produced on a band order of Blue-Green-Red (BGR) images of size 224x224 that were mean-centered across the entire ImageNet database [65], our dataset of RGB images were likewise resized and channel-wise color normalized by converting to BGR order and mean-centered by subtracting the mean of each image band using the same means computed on the ImageNet dataset (B-103.939,G-116.779,R-223.68).

First, the fully connected fusion layers of the Siamese model (Fig 3) and their weights were trained. In the first stage of training, the VGG19 convolutional weights, in both branches, and their resultant feature maps were held constant while the fully connected fusion layers were trained (Glorot normal initialization). To avoid overfitting and for computational tractability, mini-batch training was employed, and three dropout layers were added to the fully connected layers.

All training was completed using mini-batches of 4200 image pairs from the training set with image augmentation of each mini-batch. For each pair, within each mini-batch, both images had the same random augmentation applied. An image augmentation consisted of a random image warp [68] in some combination of rotation, height and width shift, zoom, shear and horizontal flipping within pre-specified thresholds. The augmentation was the same for the left and right images. Augmentation was used to avoid overfitting and to ensure that only the most relevant features were reinforced within the CNN during training [68].

To complete the training of the fully connected layers, eight augmented mini-batches were produced. For each of the eight mini-batches, the model was trained using stochastic gradient descent and 50 epochs with a batch size of 24 for each iteration. After training the fully connected layers (SCNN-FC in Table 1), the top four convolution layers were fine-tuned together for a further eight mini-batches (SCNN-FC-4 in Table 1). Finally, four mini-batches were used to fine-tune the fully connected layers and top eight convolution layers in the branches of the VGG19 Siamese model (SCNN-FC-8 in Table 1). Because the training dataset was imbalanced in favor of negative cases, class weights were employed to balance model parameter weight penalties during training. The class weights were representative of the overall dataset with 10% importance assigned to negative cases and 90% importance assigned to positive cases. Training time was approximately 33 hours on a single NVIDIA 1080ti GPU.

The final test accuracy of the fully trained model SCNN-FC-8 (~235 M trainable parameters) was 95.6% (Table 1), an AUC of 0.84 and an F1 Score of 0.72. All subsequent analyses presented herein use that model. To crudely assess the sensitivity of our model to the partitioning of the GSV training dataset, we repeated the entire training process as described above using a different random permutation of the dataset, that was subsequently partitioned into training, validation and test arrays. This second model achieved a final validation accuracy of 95.2% and test accuracy of 94.4%, an AUC of 0.82 and an F1 Score of 0.66 (Section E in S1 Appendix).

### Detection and mapping

**Fig 4:**
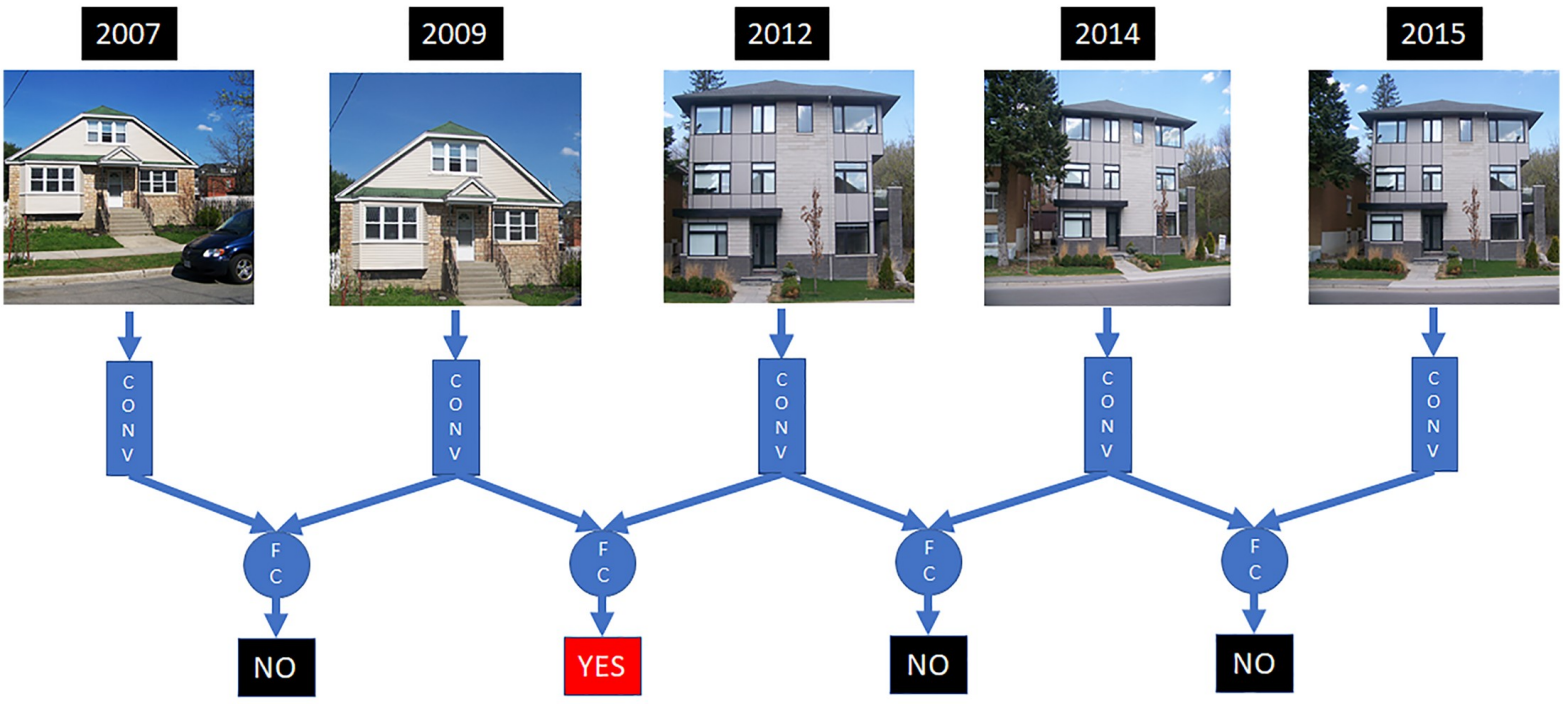
Example of detecting gentrification-like change within a sequence of GSV images at the same geographic location using SCNN-FC-8. In this example, the GSV image from 2007 and 2009 are input into the SCNN-FC-8 and the model detects whether there is a gentrification-like visual change. Only the images between 2009 and 2012 are detected as exhibiting a gentrification-like visual change in the sequence of GSV images. Each detection is recorded along with the geographic coordinates (s_x,y_) of the sequence for subsequent mapping. In order to comply with CC-BY copyright, these photos are the author’s and are not GSV imagery and so are provided for illustrative purposes only. Figure with the GSV sequence for this example can be found here: https://github.com/laggiss/DeepMapping/blob/master/GSVFIGS/fig4_online.png.

Using SCNN-FC-8, we detected on channel-wise normalized GSV image sequences at 86110 unique locations (these locations contained 403216 images in total) within the urban extent of Ottawa that contains the oldest building stock (area within the Greenbelt (Fig 1)). A single example of the detection process is illustrated in Fig 4. From the detected set, we retained those locations (points) that were identified as positive for a gentrification-like visual change as well as the year at which the change was completed. The positive detections and their location were converted to point features and used in Kernel Density Estimation (KDE) to produce mapped results.

SCNN-FC-8 will detect a gentrification-like visual change between successive GSV images whenever and wherever they occur and cannot judge whether such changes are a consequence of a *bona fide* gentrification processes or regular property maintence/home improvement. There are many types of gentrification-like visual changes that can occur to individual structures that, when in relative spatial isolation, are not associated with a potential gentrification process, despite mimicking the range of visual changes in the training dataset that we associate with gentrification for individual properties. These are visual analogues of gentrification-like visual changes. So, the model will detect gentrification-like visual changes across the spatial domain of study wherever they arise. We stipulate, however, that such changes must be numerous and occur in relatively close spatial proximity in order to define a potential gentrification process. Therefore, we are concerned with identifying the pattern of the local spatially varying intensity, *λ*, where *λ* = *n*/*A*, the number of locations, *n*, divided by the area, *A*. We used Kernel Density Estimation (KDE) with a Gaussian kernel and bandwidth of 200 m to produce maps that illustrate the spatially varying intensity of gentrification related property improvements within the study area. We used the ‘density()’ function within the R package Spatstat 1.56–0 [69] to produce the KDE estimates.

The use of KDE does not imply anything about the continuity/discontinuity of the underlying gentrification process: KDE is used to identify the likely ‘hot-spots’ of gentrification-like visual changes that are a product of the underlying spatial process of gentrification.

### Independent validation

To assess the reliability of the deep mapping approach to detect the property improvements that are visually suggestive of gentrification, we compared the mapped results from SCNN-FC-8 to the spatial distribution of building permits in the City of Ottawa from 2011 to 2016. We used the same Kernel Density estimation procedure described above to map the building permits. These permits do not account for unlawful and/or non-permitted work that takes place but represents the best independent direct measure of changes to individual structures, some of which are expressed visually—depending on the nature of the permitted work. Moreover, not all aesthetic upgrades to a property including landscaping, painting, changes to the façade (siding etc.) require building permits. Within the study area, there were a total of 56,269 permits containing 22,631 non-standardized unique descriptions of the permitted works. Because SCNN-FC-8 can only assess a property as seen from the street, the descriptions of each permitted work were used to prune the dataset to a total of 3986 permits that would most likely produce a visual change that would be viewable from the street (Section D.2 in S1 Appendix).

## Results

**Fig 5:**
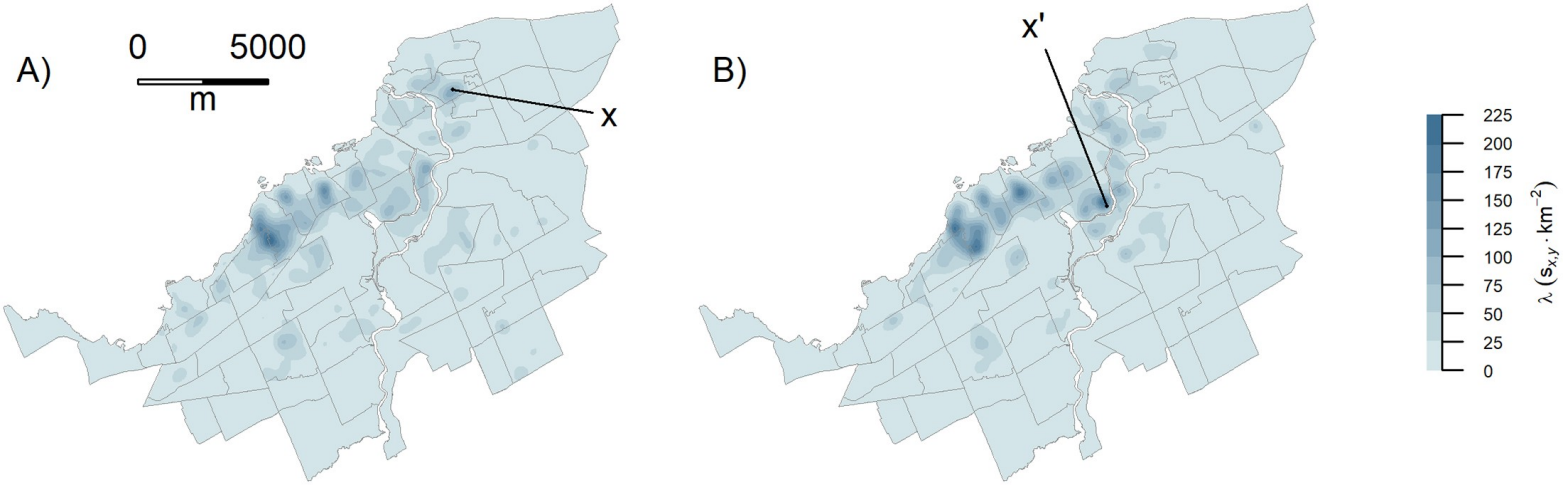
Comparison between model detections and building permits. A) Kernel density surface of SCNN-FC-8 detections; B) KDE of building permits. Both panels represent data from 2011 onwards. See text for the explanation of the labelled locations (x is within Vanier North neighbourhood and x’ is within a neighbourhood named Glebe-Dow’s Lake). Also see Section D.3 in S1 Appendix for furthur comparisions.

SCNN-FC-8 detected 3483 instances of gentrification-like visual changes at a total of 2922 unique locations. When multiple visual changes were detected for a property, we retained the most recent date when producing KDE maps. The KDE maps of the detected results and the building permits exhibit very similar patterns (Fig 5). Two notable differences in the patterns are labelled in Fig 5. The first difference (labelled x Fig 5A) is due to false positive detections that were identified because of a change in the GSV camera, whereby the images along one street were offset for the same geographic locations between 2007 and 2009. The second large difference (labelled x’ on Fig 5B) represents permits within a multi-hectare redevelopment of the city’s ageing cattle dome/football stadium, the majority of which could not be seen in GSV. A difference map of the standardized intensities highlights both x and x’ as spatially distinct regions of differing intensities between the KDE of visual changes detected by SCNN-FC-8 and the KDE of building permits (Figure F(A) in S1 Appendix).

**Fig 6:**
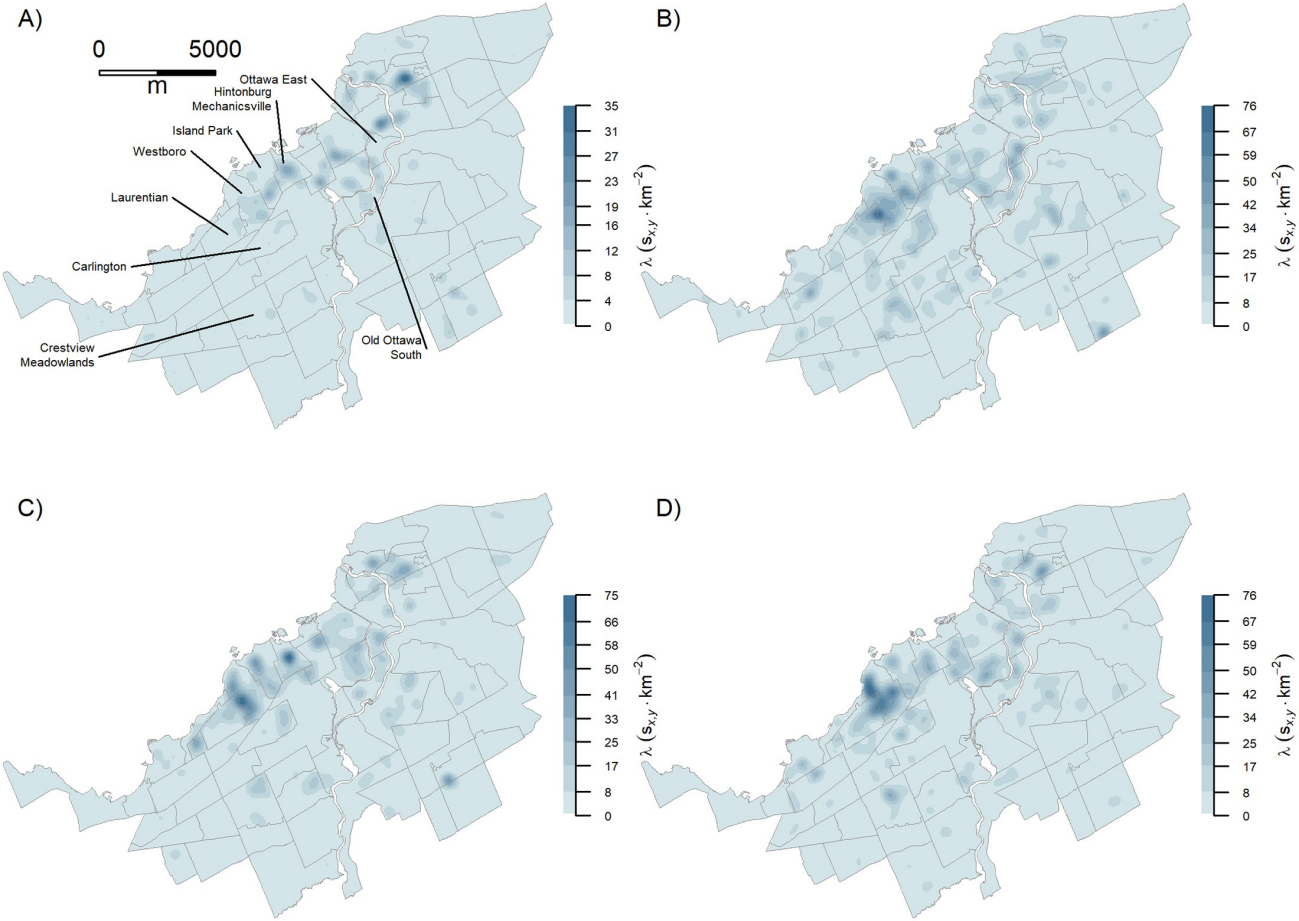
SCNN-FC-8 detections over time. A) 2007–2009—with select neighborhoods labelled; B) 2009–2012; (c) 2012–2014; (d) 2014-2015/16.

KDE maps can be produced within the study area at intervals spanning 2–3 years as defined by the update interval of GSV images (Fig 6). Because of sparse GSV coverage in 2016 (Figure D in S1 Appendix), we combined all the positive detections for 2015–2016 (Fig 6D). Changes which are taking place can be seen in areas which are typical for gentrification in classic literature—inner city areas which are close to amenities and which have pleasant architecture. For example, the region of high intensity that is occurring between the Westboro and Laurentian neighbourhoods exhibits a shift northward, locally, in intensity between 2012–2014 and then expands appreciably between 2014–16 (Fig 6). However, some areas within the more established regions such as Crestview-Meadowlands, Island Park and Ottawa East are also identified as having regions of high intensity of gentrification-like visual changes to properties from 2012 onwards (Fig 6).

## Discussion

Despite the pervasiveness of gentrification in modern cities, the focus on social, economic and cultural discourse around the phenomenon has led to a neglect in the development of methods that quantify the temporal and spatial evolution of the phenomenon itself. Typically, to identify gentrification, census data is analyzed over time to identify socioeconomic changes in census tract structure [11,50,70,71]. However, as our maps illustrate, gentrification-like visual processes are often localized, particularly in the initial stages and have no natural respect for artificial census boundaries. In general, census data places restrictions on the ability to quantify the process of gentrification at arbitrary temporal and spatial domains.

Spatially, census data implicitly assumes that variables are homogenous within the census unit. Thus, the lack of within-census unit variation limits the spatial resolution of analysis and interpretation to the scale of the census unit. Moreover, because census units are artificial spatial units (e.g., census tracts), interpretations are confounded by the modifiable areal unit problem’s (MAUP) analytical effects of zoning and scale [72]. By way of illustration, if a bona fide gentrification process spans several census unit boundaries, there may not be enough compositional changes in key census indicators within any one of the spatial units to indicate that a gentrification process itself has taken or is taking place, increasing the chances of false negatives when mapping gentrification. Consequently, the delineation of gentrifying or gentrified areas becomes ambiguous [35]. Temporally, the frequency at which gentrification can be gauged from census data is at five- to ten-year intervals (in Canada and the U.S.). As such, gentrification may often be detected only after it has progressed to a large extent within census tracts. To study the gentrification process spatially, optimally, requires being unconstrained from the spatial and temporal limitations of census data by precisely locating when and where the process is within geographic space.

Our results show that a deep mapping approach centered on the sequence of GSV images of an individual property is successful in spatially resolving likely gentrification processes within urban areas and has potential, upon detailed analysis of the GSV data, to provide a high spatial and temporal resolution that could be of benefit in testing theories of urban renewal. For example, to test the invasion hypothesis, Naik et al. (2017) aggregated their Streetchange and Streetscores to census tracts and showed that a neighborhood spillover effect was present by establishing that the Streetscores of adjacent neighborhoods increased the Streetchange of a given neighbourhood positively through time. Our KDE results potentially allow for the decomposition of spillover effects over time. Recall, for example, that our results detected that the center of high intensity between the Laurentian and Westboro neighbourhoods in 2007–9 moved both north and south along the neighbourhood boundary by 2014–2016 (Fig 6). Thus, by focusing on the property as the unit of comparison, we can see a potential spillover effect within the KDE results. However, that being said, we cannot rule out that the observed adjacent and sequential clusters are not due to some spontaneous influence.

**Fig 7:**
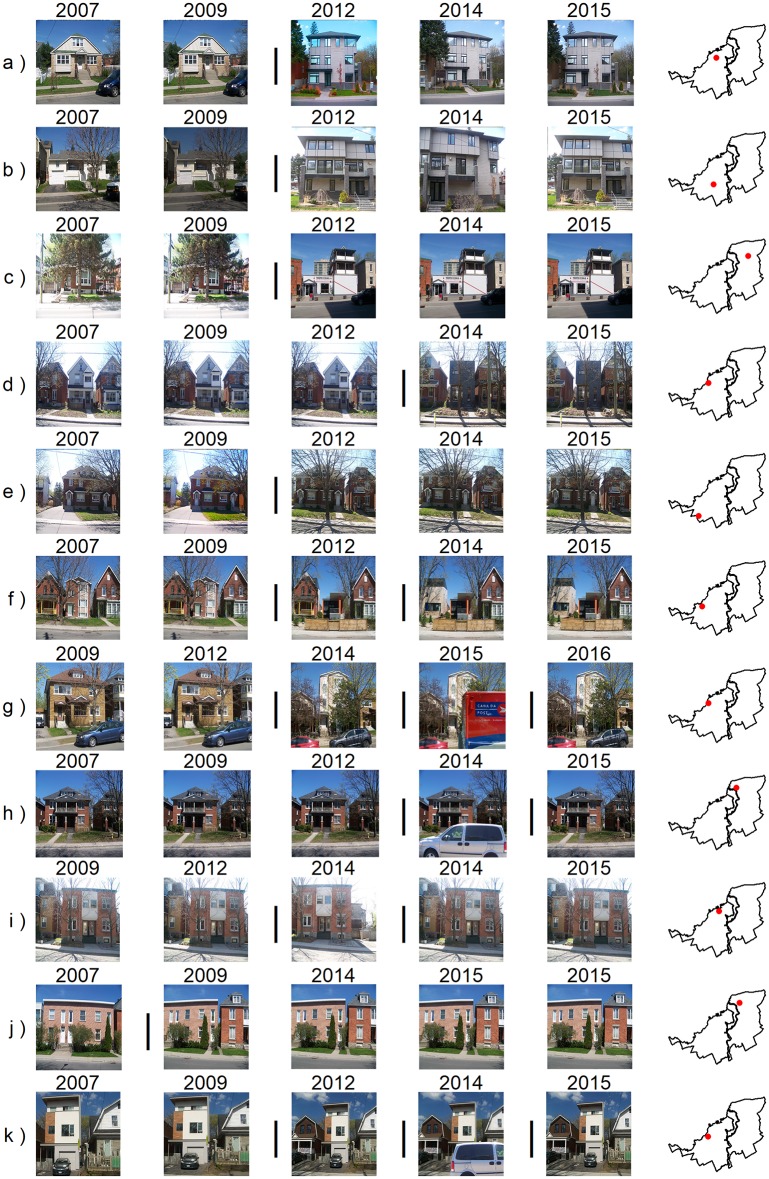
Examples of SCNN-FC-8 detections. The photos here are the authors’ and have been manipulated for illustrative purposes. In order to comply with CC-BY copyright, a figure containing detected changes within GSV image sequences can be found in this research project’s GitHub repository at: https://github.com/laggiss/DeepMapping/blob/master/GSVFIGS/fig7_online.png. The reader should refer to the external figure because it fully captures the descriptions in the text. Each row represents the time series of GSV images for the unique location specified in the map plot at the end of each row. The vertical bar (׀) symbol between any two successive years indicates that the SCNN-FC-8 detected a visual gentrification-like change between the two GSV images. (a to d) Normal cases of a single change being detected across the series of images at a given unique location. (e to k) represents cases where the model detected more than one change or detected a false positive change within the sequence for some other reason as detailed in the text.

In general, our results indicate those areas that contain re-investment in the housing stock as exhibited by a progressively upward improvement of aesthetic appearance of properties that is typical of gentrification (Fig 7).

When SCNN-FC-8 examined a sequence of GSV images for a property, most of the time only one instance of gentrification-like visual change was found. Typical visual features found when a single instance of change was detected, include an older structure being torn-down and a new more expensive structure being created (Fig 7A to 7C) or a significant change in landscaping and/or façade (Fig 7D). Fig 7A to 7D also illustrate that the model is quite robust to the removal of a structure, vegetation phenology, varying sky/cloud conditions and exposure. The robustness of CNN models with respect to variations in the photographic quality and weather variations equate to less image pre-processing when compared to some other machine mapping studies like that of Naik et al. (2015, 2014, 2017) who, for example, had to control for trees and sky when using feature extraction and support vector regression in order to detect semantic qualities of GSV images.

SCNN-FC-8 detected approximately 16% of the properties as containing more than one instance of a gentrification-like visual change: 399 properties showed two instances of change, 57 showed three instances of change and 16 showed four instances of change. When capital is invested in the built environment, it is also immobilized for varying lengths of time [73]. Developers and investors aim to minimize housing production and holding costs, and from that perspective, multiple major upgrades or redevelopment to property over several years should generally be infrequent to maximize returns on investment.

In cases where multiple detections of visual change were found at the same location, there were several reasons. Foremost, most false positive detections occurred when the camera location was either offset or had a considerably different field of view (FOV) in a subsequent GSV at the same location—the coordinates that were sent to the Google API. This coordinate offset/FOV meant that a GSV image in at least one year had the camera pointing either between two structures, whereas previously it was pointed at one structure or *vice versa*. The sudden appearance of a second structure entering the GSV image led to SCNN-FC-8 incorrectly identifying a gentrification-like visual change (Fig 7E, 7I and 7J). When such effects occurred, examination of the activation patterns of the last convolutional block tended to show strong activation on the neighboring structure (Section F in S1 Appendix). These offset/FOV-induced false positive detections were pronounced in at least one neighbourhood (Vanier North) (Fig 5A). SCNN-FC-8 was robust to smaller changes in offset/FOV (Fig 7A to 7D). A common issue with using GSV images in deep mapping is obfuscation of the subject by a large vehicle [50]. If a large vehicle was obscuring a property in one image and gone in the next, the model would detect a false positive gentrification-like visual change (Fig 7G, 7H and 7K). Such objects tended to be strongly activated upon in the last convolutional block causing the differences in the visual appearance of a property to force a positive decision by SCNN-FC-8, even though no gentrification-like visual change was evident. In other cases, multiple-detections at the same location were not false positive detections but were due to multi-year construction periods on the property (Fig 7F, 7G and 7K). In some of those cases, more than one structure was within the FOV of the GSV image, the first instance of change was due to an adjacent structure undergoing modification that was then followed by a transformation to the second structure (Fig 7G and 7K). In these cases, our model would detect multiple changes to a property beginning with the major structural change and followed by subsequent updates to the new façade if construction spanned more than one year or one property. Because two instances of change occurred in 399/472 cases of multiple change that were detected by SCNN-FC-8, we retained only the most recent date at which a gentrification-like visual change occurred when producing our maps. Improvement of properties was not limited to residential properties. Our model also detected commercial gentrification, which can be observed through the improvement of store fronts and significant upgrading to streetscapes.

To understand the emergent pattern produced by the model detections we used Kernel Density Estimation (KDE). When there are spatial clusters of SCNN-FC-8 detected changes that occur within historically stagnant urban areas, these changes are more likely to indicate a gentrification process occurring locally. Because our interpretation of gentrification relies heavily on the spatial intensity of gentrification-like visual changes, local or regional disasters could lead to renewed property reconstruction and regions of high intensities of gentrification-like visual change that are not driven by gentrification. However, in Ottawa during the period of study, there were no large-scale disasters that would have induced local clusters of property reconstruction.

Our reliance on KDE to identify hot-spots of gentrification-like visual change assumes that there is a background intensity of physical property improvements that are non-gentrification related and follow an inhomogeneous Poisson process. It would be reasonable to assume that this inhomogeneous process is random through space and time contingent on the distribution of structures in a city and secondly their age. Our current mapping process includes the unknown background intensity estimate within the KDE maps. However, because the full GSV dataset within the study area is already contingent on the spatial distribution of building stock, a spatially random process contingent on that building stock would be unlikely to produce the high-intensities we find on our KDE maps of the model detections. In other words, by focusing on intensity as the basis for inference about the gentrification process, we can ignore the background noise because spatially isolated properties exhibiting gentrification-like visual changes contribute little to the intensity surface at any location. Further research is required to understand and estimate the background process of gentrification-like visual changes within a city which, when controlled for, would lead to more accurate and focused spatial estimates of intensity.

The validation of SCNN-FC-8 showed that the spatial patterns of our model and that of permits were strikingly similar (Fig 5)—with a few minor variations due to false positives in the model detections. When a permit is issued, GSV images at the permit location might show an improvement that same year. Sometimes the improvement might appear only a year or two later, due to the month in which the permit was issued and the length of time necessary for construction. This lag time would partly explain minor differences observed between the KDE maps of permits and the model detections. Other differences may be in part due to the criteria for retaining permits. It was important to differentiate suburbanization from gentrification, as these are clearly different processes. When examining permits, “new build gentrification” was retained. This type of new construction might not be picked up by our model and can be a contentious matter in terms of whether or not such developments represent gentrification at all [74,75]. Finally, in some cases, a region of high intensity in SCNN-FC-8 detections could be due to false positive detections—as was the case in Vanier North (Fig 5) caused by offset/FOV differences between sequential GSV images along one street. In such cases, KDE shows regions of high intensity that would be greater than the background noise. Without careful examination of the KDE results, such false-positive regions could be attributed to a gentrification process.

Our KDE maps of gentrification-like visual changes agree with the recent accounts of gentrification in the City of Ottawa. The greatest intensity of gentrification-like visual changes were detected in the region of Westboro/Laurentian followed by Hintonburg/Mechanicsville, both of which are local gentrification hot-spots in Ottawa [76–79]. In addition, we found high intensities in regions we did not know were undergoing gentrification like the neighbourhoods of Crestview-Meadowlands, Island Park and Ottawa East among others. While we have noted the issues in Vanier North, some of the detections were valid and agree with observations of urban densification and new build gentrification over the past decade in that neighborhood [80]. Similarly, we find moderate intensity in Old Ottawa South, a neighbourhood that contained gentrification largely prior to our study [81], but just across the northern boundary we found Ottawa East to be currently undergoing intense gentrification.

A distinct advantage of using a deep mapping approach based on CNNs, at least in our case, is the robustness of the model with respect to GSV images containing different weather, exposure and vegetation phenology. However, we worked in a restricted spatial domain and expect that false-positive model detections will be, for the most part, contained in the random background. Still, using a larger GSV dataset for detection could lead to areas of high intensity of gentrification-like visual changes being identified that are due to weather or exposure issues. With more expansive GSV datasets it becomes increasingly important to have examples of all possible GSV image conditions represented within the subset of data used for training.

## Conclusion

By taking a deep mapping approach to detecting gentrification, we have shown that it is possible to indicate precisely where and when gentrification processes are happening in an urban area. By focusing on the detection of gentrification-like visual changes to individual properties, SCNN-FC-8 results could be aggregated to any arbitrary geography and thereby specify the proportion of any arbitrary spatial unit that has gentrified. One advantage of doing so would be to test scale and zoning effects of the MAUP on results that are tied to a set of artificial boundaries (such as census tracts or neighbourhoods). For example, with SCNN-FC-8, mapped results are able show if two blocks gentrify around a boundary. This could aid in validating or decomposing the results of census-based inferences about gentrification in urban areas. While the SCNN-FC-8 model was developed and tested in the context of Ottawa, we believe that similar results can be reproduced in other urban contexts within peer countries. It remains to be seen what or if similar results can be replicated in different contexts and the degree to which we can detect different kinds of gentrification (such as slum gentrification). Definitions and visual indicators of gentrification can differ based on cultural or architectural norms across different countries. With a relatively small but regionally consistent training dataset, other north-American locales would be able to train the SCNN-FC-8 architecture using a transfer learning approach.

Given the relative ease with which deep learning models can now be applied, thanks to high level APIs like Keras [61], a relatively modest time investment can produce highly spatially and temporally resolved maps of the gentrification process. Defining the rate and location of gentrification-like visual changes can directly benefit municipalities at a number of levels: the tax-assessor can use the information to model future property tax income scenarios; city budget and planning can use detailed maps of gentrification in order to prioritize infrastructure upgrades; the transportation office can determine where traffic-calming measures or bicycle lanes might be required in rapidly gentrifying areas; the building permit office could examine results to aid in locating non-permitted work; and, city zoning can use the information in re-zoning application reviews; in the current neoliberal era, public housing is ever scarcer and the mapping of gentrification could indicate areas in need of assistance in response to displacement. An investor could speculate on where to invest within a city by examining where gentrification is happening and where the likely spill-over will occur. Finally, from a competition perspective, retail location analysis would benefit significantly by establishing new locations where gentrification is happening or ensuing.

## Supporting information

S1 Appendix Supplementary information for ‘Deep mapping gentrification in a large Canadian city using deep learning and Google Street View’. Figure A. GSV panorama distributions used in this research. Figure B. Frequency distributions. Figure C. Spatial distribution of properties that have a panorama in 2007 and also in 2015–2016. Figure D. Spatial distribution of all accessed GSV images through time. Figure E. Training and validation of SCNN-FC-8 (Epoch 1 to min(loss)). Figure F. Comparison of KDE maps. Figure G. Comparison of A) SCNN-FC-8 results with B) the replicate model results.
(PDF)
