# Supplementary material for: Deep mapping gentrification in a large Canadian city using deep learning and Google Street View
Source: PLoS One. 2019 Mar 13;14(3):e0212814. doi: 10.1371/journal.pone.0212814 (PMC6415887; doi:10.1371/journal.pone.0212814)
Supplement: S1 Appendix — Figure A. GSV panorama distributions used in this research. Figure B. Frequency distributions. Figure C. Spatial distribution of properties that have a panorama in 2007 and also in 2015–2016. Figure D. Spatial distribution of all accessed GSV images through time. Figure E. Training and validation of SCNN-FC-8 (Epoch 1 to min(loss)). Figure F. Comparison of KDE maps. Figure G. Comparison of A) SCNN-FC-8 results with B) the replicate model results. (PDF) [file pone.0212814.s001.pdf]

S1 Appendix

Supplementary information for 'Deep mapping gentrification in a large Canadian city using deep learning and Google Street View'

Lazar Ilic, M. Sawada, Amaury Zarzelli

Contents

A Introduction **1**

B Github repository **1**

C Study area and GSV data **2**

D Results **5**
D.1 SCNN-FC-8 model training and validation 5
D.2 Building Permits 5
D.3 SCNN-FC-8 and building permits 8

E SCNN-FC-8 sensitivity **9**

F Activation maps **10**

A Introduction

In the main manuscript we describe the use of SCNN-FC-8 to detect gentrification-like visual changes within a sequence of GSV images for a given property. This document contains supplementary explanations and figures that elaborate upon information in the main manuscript.

B Github repository

The set of python scripts used to create and train SCNN-FC-8 can be found at:

*https://github.com/laggiss/DeepMapping

> **NOTE**: *This document was created from an RMarkdown file. The RMarkdown version of this doucument contains all of the R-language code used to access datasets as well as create the maps (except Fig 1 in the manuscript which was created elsewhere) and graphs in this supplementary document and manuscript. However, the R code is suppressed in the version you are currently reading.

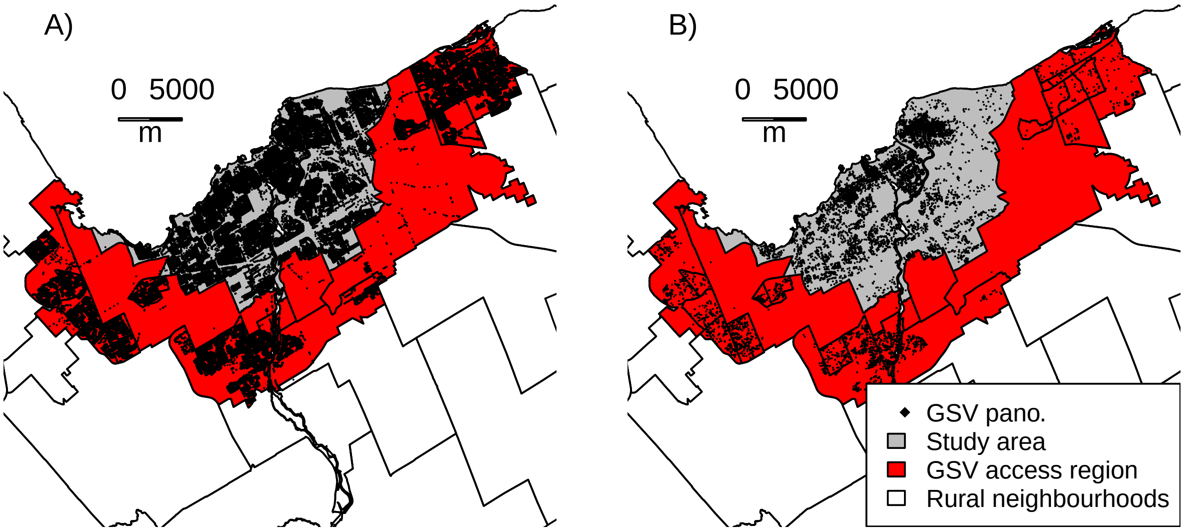

Figure A: GSV panorama distributions used in this research. A) Distribution of all GSV panormas accessed in this research; B) Subset of GSV panoramas within the GSV access area that were used in training,validation and testing of SCNN-FC-8.

Should you wish to examine the raw data or code, you can find the RMarkdown document with R-language code containing all references at: https://github.com/laggiss/DeepMapping/blob/master/sampling.Rmd

C Study area and GSV data

The spatio-temporal coverage of GSV imagery varies across space and time. We were primarily concerned with detecting gentrification as far back as possible within our 'study area'. The study area corresponds to the spatial domain defined by the highest concentration of older building stock in Ottawa (see Fig 1 in manuscript). To train SCNN-FC-8, we wanted a larger region in which to find examples of gentrification-like visual change. Thus, we accessed GSV imagery within a broader region that includes our study area and a buffer region that, itself, is comprised primarily of post 1980's suburban residential land use. We call this broader region the 'GSV access region' within which GSV panoramas were selected to train SCNN-FC-8 (Fig A(A)). There were 157303 properties containing panoramas within the GSV access region. Because each property/location can have numerous panoramas over time, the total number of panoramas across the properties was 593,723. A subset of 16224 GSV images were clipped fronm the panoramas to extract individual buildings by sending the field of view (FOV) and camera direction to the Google Maps API. That subset was used for training, validating and testing SCNN-FC-8 (Fig A(B).

Next, we determine if the panoramas within the GSV access region can support analysis in

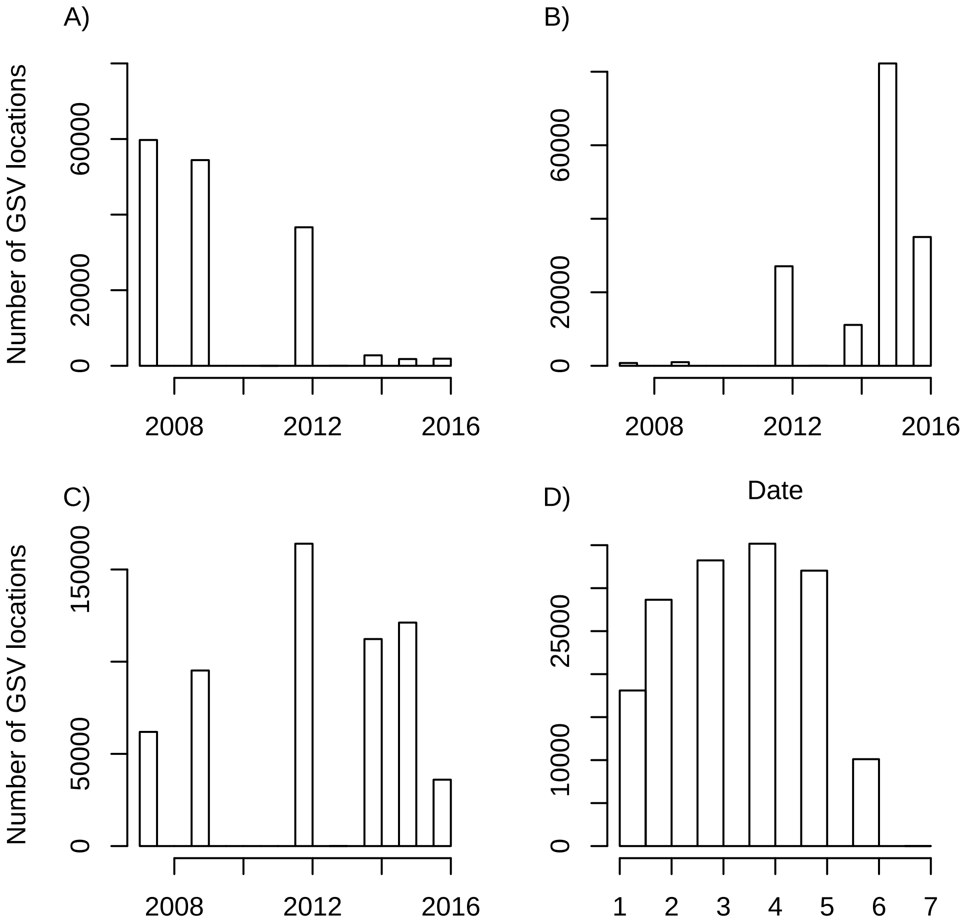

Figure B: Frequency distributions. A) Distribution of the oldest GSV date accessed at a location; B) Distribution of the Youngest GSV date accessed at a location; C) Distribution of all GSV image dates accessed at a location; D) Distribution of the number of years of GSV imagery at each queried location.

the study area back to 2007. The first GSV panoramas in the city were taken in 2007 and we would like to map changes as far back as possible when describing the spatial evolution of gentrification in the study area. Thus, the possible temporal domain for this study is from 2007-2016 (we accessed imagery for this work in the spring of 2017). However, GSV images may not be available at all time periods at all locations. In addition, we also also determine which years in the interval between 2007 and 2016 contained sufficient spatial coverage.

Within the GSV access region (Fig A), the majority of properties had GSV images by 2012 (Fig B(A)) and the majority of these were fully re-imaged between 2014-2016 (Fig BB). 2012 is the year with the most panoramas (Fig B(C)). Fig B(C) provide evidence of an approximate 2-3 year update frequency of GSV imagery. The average number of years that GSV panoramas are available at a property is 3.41 Fig B(D).

Examining all properties (locations) that contained an image in 2007 and one in the years

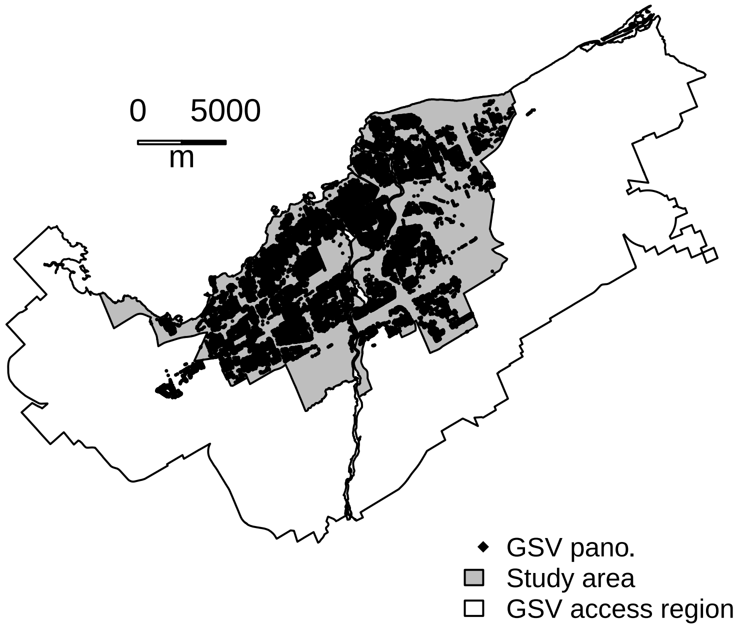

Figure C: Spatial distribution of properties that have a panorama in 2007 and also in 2015-2016.

2015-2016 , most clearly defines the region in which we can make inferences back to 2007 (Fig C). We cannot, for example, make inferences regarding changes in the spatial distribution of gentrification-like visual changes between the years 2009-2010, since in 2010 there are almost no GSV panoramas.

Concerning the spatial distribution of GSV images over the study period and within the study area, we examined the spatial distribution of the GSV panorama dates through time. In Fig C, it is apparent that only the study region contains sufficient spatial coverage back to 2007 (Fig C). Google did not expand coverage into the GSV access region until 2009 in Ottawa. The study region is completely covered with panoramas in 2007,2009,2012,2014, and 2015 (Fig D). Therefore, we were confident in using SCNN-FC-8 to detect gentrification-like visual changes within the study region. Regarding the examination of gentrification through time, we can compare GSV images within the study area between the years: 2007-2009,2009-2012,2012-2014 and between 2014 and a combined 2014-2016 (Fig C).

D Results

D.1 SCNN-FC-8 model training and validation

D.2 Building Permits

The City of Ottawa building permits are organized by month and by year in excel files and each permit contains fields such as address, date of issue and the description of work as well as the cost. We concatenated permit lists from July 2011 through December 2016 which produced a list of 56,269 permits that had 22631 unique descriptions. This total was reduced, as is explained in the following.

Through permits' descriptions it was possible to trim the list by removing those which were deemed redundant, as well as to establish a list of relevant permits. After pruning the permit list, 1391 permit descriptions remained and were inspected manually, after which the total number of permits amounted to 27,509.

After removing permits belonging to structures outside the study area, 6,342 permits remained. Each remaining permit location was manually inspected on Street View, after which the list was further reduced to a final number of 2356. There were two criteria for retaining permits. The first was to only keep permits whose addresses can be seen on Street View, and the second was that the change observed to the property of the permit must have underwent a change which yielded a progressively more affluent structure. Some examples can include the addition of a car port, additions to the side of the house, or the construction of a completely new structure. However, more permits were removed (3986) than were kept. This is not to imply that the removed permit entrees are of structures which were not improved, but rather that their improvements are not visible from the street. Rear additions, basement alterations, and foundation repair are examples of improvements which do not necessarily yield a visual change.

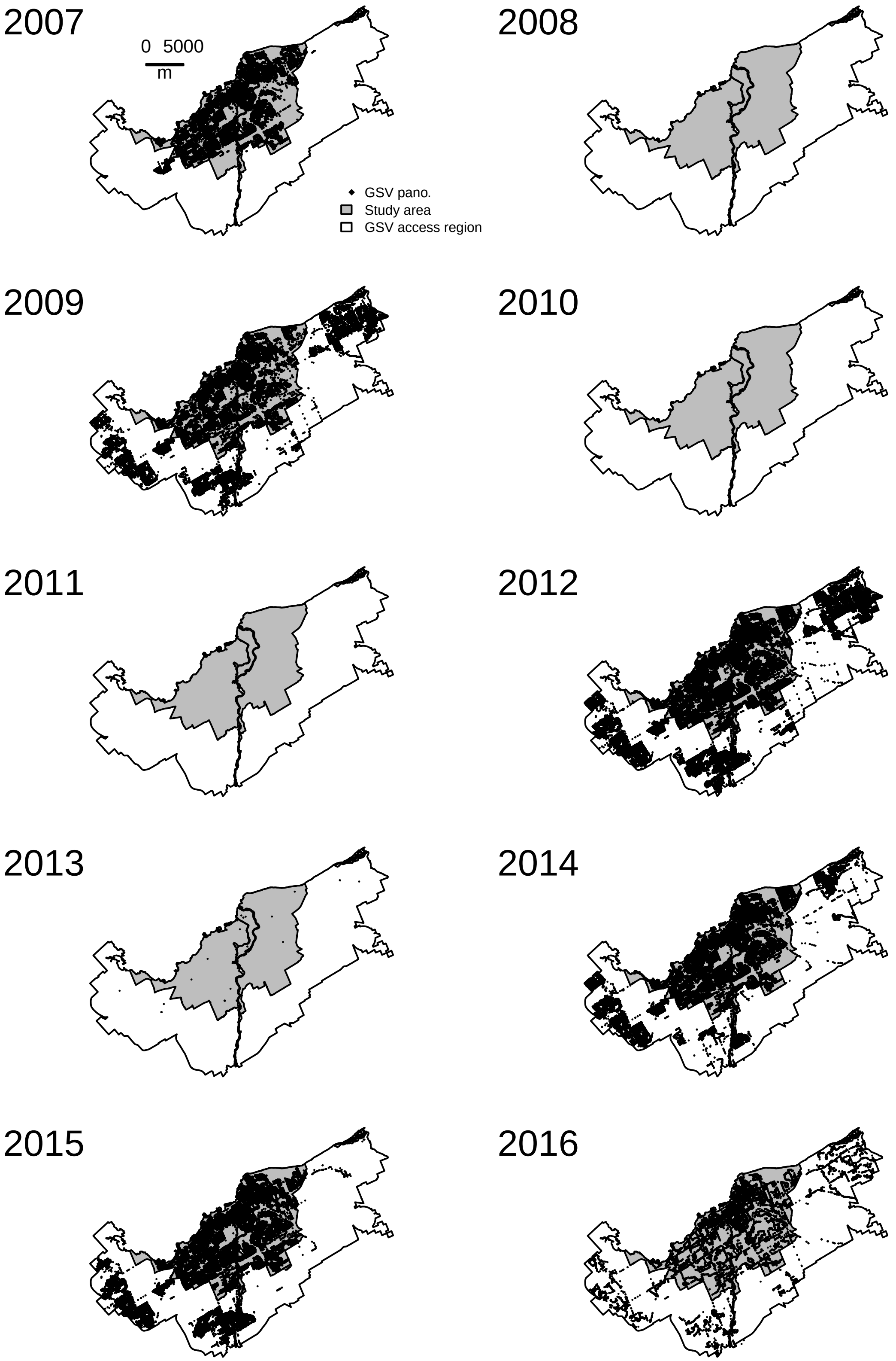

Figure D: Spatial distribution of all accessed GSV images through time.

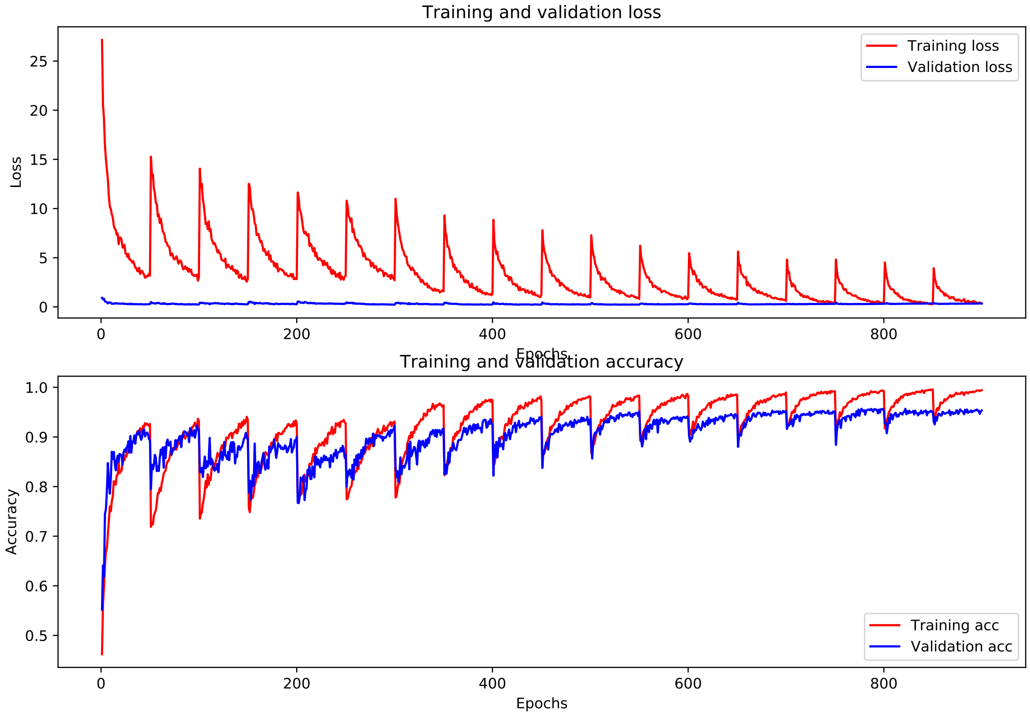

Figure E: Training and validation of SCNN-FC-8 (Epoch 1 to min(loss)).

Street View lacks imagery on certain streets and segments of streets. This is most common with suburban subdivisions which are designated as privates. Some permits were deleted because their addresses were obfuscated on Street View. Other permits were deleted as the upgrades that they represented are not indicators of gentrification. This would include certain commercial infrastructure such as: industrial buildings, warehouses and storage structures. Institutional infrastructure was also removed, and included objects such as universities, schools, fire stations, churches, hospitals, community centers and transit infrastructure.

The SQL statements used to refine the permits can be found here: https://github.com/laggiss/DeepMapping/blob/master/sql_statements_permits.txt

D.3 SCNN-FC-8 and building permits

We could have used KDE for point patterns based on a network *c.f.*[Okabe and Sugihara, 2012]. However, strictly speaking, because the spatial support for the points relate to an area unit, the property, our point data violates the basic assumption of point pattern analyses, either in free space or on a network, both of which requires points to be representative of discrete entities. That being said, because the point locations (x,y) are a dimensional reduction of the property polygon, we use KDE on what is effectively a point process that contains dispersion at small scales - because the property areas are approximately equal. Our choice of bandwidth is much larger than the small scale dispersion among adjacent properties and so a valid way to visualize and compare patterns is by using KDE. We are interested in the location and scale of the gentrification process as defined by the clustering of gentrification-like visual changes.

We found the pattern of building permits and SCNN-FC-8 predictions over the same period to be very similar, with only a few minor deviations (Fig 5 in Manuscript).

Fig F(A) illustrates the difference between the standardized intensity, λ, of the SCNN-FC-8 KDE prediction map and KDE of building permits. The two isolated regions of intensity in Fig F(A), x and x', clearly stand out as the two largest differences between the model predictions and the building permits. As discussed in the main text, x' is due to a large development of Landsdowne Park (https://en.wikipedia.org/wiki/Lansdowne_Park_redevelopment), and x was due to false positive detection's of gentrification-like visual change because of offset/FOV issues. In particular, in the region denoted by x', there were a number of new homes constructed which normally would have been detected by SCNN-FC-8 within the image sequence. However, after examining the sequences of GSV images in the region of x', a common theme became apparent in the sequence of visual changes: Initially, a park was present and that was followed by construction of a large wall. The wall was eventual removed and a new house was constructed. The change from a wall to a residential structure, or from a park to wall for that matter, was not something that SCNN-FC-8 encountered in our training data set. Thus such changes were not reinforced in training as being gentrification-like instances of visual change.

Fig F(B) represents the local Pearson's correlation coefficient between the standardized intensity {lambda} of the SCNN-FC-8 KDE prediction map and KDE of building permits.

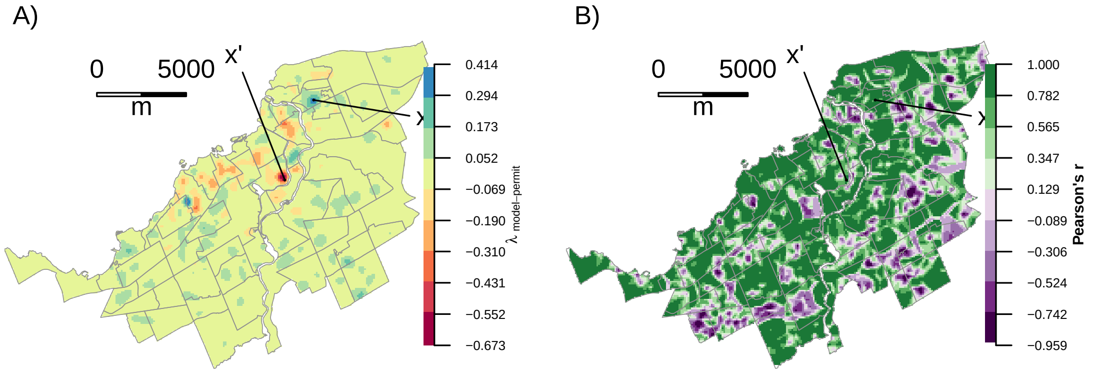

Figure F: Comparison of KDE maps. A) Proportional difference betweeen predicted KDE and permit KDE. B) Local Pearson's R between predicted KDE and permit KDE.

We use the correlation coefficient **r** as an exploratory tool to identify local regions of similarity/dissimilarity in intensity between the model predictions and the permits. This map represents the correlation coefficient in a 5x5 moving window between the standardized values from manuscript Fig 5AB. The large amount of green area in Fig F(B), indicates that the majority of the intensity values strongly covary, which is evident in the visual inspection of Fig 5AB (Manuscript). There are strong dissimilarities or negative correlations locally, but these are generally in areas of very low spatial intensity around the southern boundary of the study region. In other words, the majority of disagreements in the model/permits comparison are in regions where there are few gentrification-like visual changes. This further reinforces the concordance seen between SCNN-FC-8 and the building permits upon visual comparison of their mapped intensities.

E SCNN-FC-8 sensitivity

After training SCNN-FC-8 as described in the manuscript, we wanted a crude test of how different our results would be if we reshuffled all of the training data and re-trained the model from scratch (the model training procedure is described in the manuscript). The training data set contained all 16224 GSV images and we used 60% of these to train SCNN-FC-8, holding back 20% for model validation during training, and the last 20% were held back for testing the model once we achieved our desired model validation accuracy. All 16224 image pairs were shuffled/permuted prior to being split. To retrain the model once again, we simply reshuffled all 16224 GSV image pairs and reapplied the training procedure. After training this second model instance, all GSV image sequences were fed through the model and the

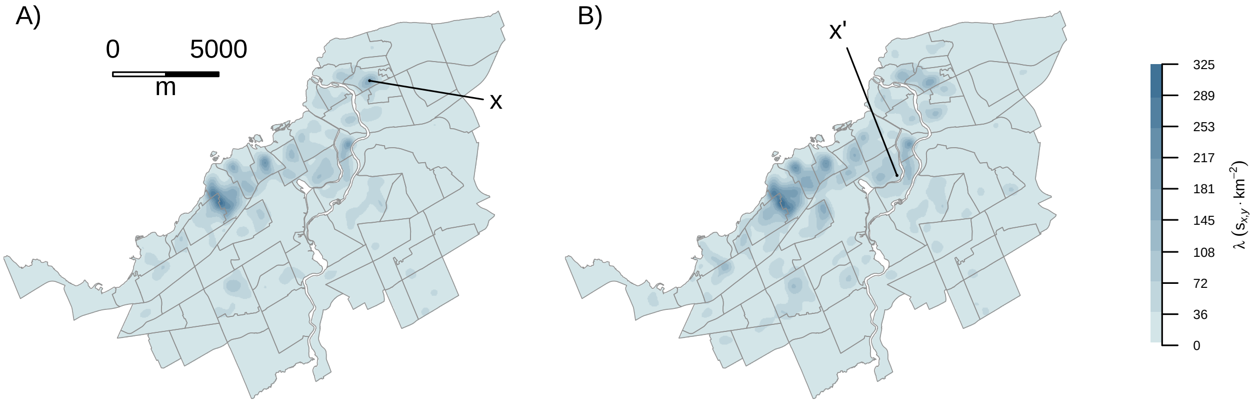

Figure G: Comparison of A) SCNN-FC-8 results with B) the replicate model results

results were mapped (Fig G). The spatial pattern of intensity between SCNN-FC-8 (Fig G(A)) and the second model (Fig G(B)) are nearly identical. This suggests that the results obtained using SCNN-FC-8 are not significantly biased by the train/validation/test split.

F Activation maps

Activation maps can be used to explore which features in a GSV image are most strongly reinforced in SCNN-FC-8. The activation maps of the last convolution block for each image in a properties sequence (Fig 8), aid in revealing the potential reasons why some false-positive detection's were made by SCNN-FC-8. Generally, we expect similar activation maps on the t[0] and t[0]+1 GSV images when no gentrification-like visual change is detected by SCNN-FC-8. Conversely, when a change is detected, there should be differences in which features are most strongly activated upon by SCNN-FC-8. The activation maps in Fig 8 -https://github.com/laggiss/DeepMapping/blob/master/GSVFIGS/figS8_online.png-correspond to the images in the manuscript Fig 7-https://github.com/laggiss/DeepMapping/blob/master/GSVFIGS/fig7_online.png. See manuscript for further explanation.

References

Atsuyuki Okabe and Kokichi Sugihara. *Spatial Analysis along Networks: Statistical and Computational Methods*. John Wiley & Sons, Ltd, Chichester, UK, July 2012. ISBN 978-1-119-96710-1 978-0-470-77081-8. doi: 10.1002/9781119967101. URL http://doi.wiley.com/10.1002/9781119967101.
